# miRNAs and sports: tracking training status and potentially confounding diagnoses

**DOI:** 10.1186/s12967-016-0974-x

**Published:** 2016-07-26

**Authors:** Anne Hecksteden, Petra Leidinger, Christina Backes, Stefanie Rheinheimer, Mark Pfeiffer, Alexander Ferrauti, Michael Kellmann, Farbod Sedaghat, Benjamin Meder, Eckart Meese, Tim Meyer, Andreas Keller

**Affiliations:** 1 Institute of Sports and Preventive Medicine, Saarland University, Saarbrücken, Germany; 2 Department of Human Genetics, Saarland University, Saarbrücken, Germany; 3 Chair for Clinical Bioinformatics, Medical Department, Saarland University, Building E2.1, 66125 Saarbrücken, Germany; 4 Department of Theory and Practice of Sports, Johannes Gutenberg University of Mainz, Mainz, Germany; 5 Faculty of Sport Science, Ruhr-University Bochum, Bochum, Germany; 6 School of Human Movement Studies, The University of Queensland, St Lucia, Australia; 7 Internal Medicine, Heidelberg University, Heidelberg, Germany

## Abstract

**Background:**

The dependency of miRNA abundance from physiological processes such as exercises remains partially understood. We set out to analyze the effect of physical exercises on miRNA profiles in blood and plasma of endurance and strength athletes in a systematic manner and correlated differentially abundant miRNAs in athletes to disease miRNAs biomarkers towards a better understanding of how physical exercise may confound disease diagnosis by miRNAs.

**Methods:**

We profiled blood and plasma of 29 athletes before and after exercise. With four samples analyzed for each individual we analyzed 116 full miRNomes. The study set-up enabled paired analyses of individuals. Affected miRNAs were investigated for known disease associations using network analysis.

**Results:**

MiRNA patterns in blood and plasma of endurance and strength athletes vary significantly with differences in blood outreaching variations in plasma. We found only moderate differences between the miRNA levels before training and the RNA levels after training as compared to the more obvious variations found between strength athletes and endurance athletes. We observed significant variations in the abundance of miR-140-3p that is a known circulating disease markers (raw and adjusted p value of 5 × 10^−12^ and 4 × 10^−7^). Similarly, the levels of miR-140-5p and miR-650, both of which have been reported as makers for a wide range of human pathologies significantly depend on the training mode. Among the most affected disease categories we found acute myocardial infarction. MiRNAs, which are up-regulated in endurance athletes inhibit VEGFA as shown by systems biology analysis of experimentally validated target genes.

**Conclusion:**

We provide evidence that the mode and the extent of training are important confounding factors for a miRNA based disease diagnosis.

**Electronic supplementary material:**

The online version of this article (doi:10.1186/s12967-016-0974-x) contains supplementary material, which is available to authorized users.

## Background

It is increasingly recognized that biomarkers are not only affected by pathological processes, but likewise by other environmental factors. Among the most popular examples are cardiovascular biomarkers such as cTnT, hs-cTnT, BNP or NT-proBNP. These biomarkers are prone to alterations due to strenuous exercise, as recently reviewed by Sedaghat-Hamedani [1]. Similarly, more complex marker signatures may also depend significantly on the training status. One class of such novel marker candidates are small non-coding RNAs, so-called miRNAs.

Their importance for a wide range of conditions is currently explored and validated. Beyond tissue based miRNA profiles, circulating patterns have gained increasing importance. The role of circulating miRNAs in different disease classes has been reviewed in depth. Examples include the two most common causes for death in developed countries, cancer [2] and cardiovascular disorders [3]. In addition to their promising role as diagnostic and prognostic markers for human pathologies, miRNAs have also been correlated to different exercise modes and the overall physical performance capacity. Especially in the light of more complex marker patterns that are not only correlated to diseases but also to physiological processes such as exercises it is essential to understand how respective physiological processes can confound disease diagnosis and prognosis.

Previously, we reported that exercise of elite endurance athletes had a limited direct influence on miRNAs in blood [4]. In the same study we reported that respective changes in the blood have lower effect sizes as compared to the impact of pathophysiological changes. In other studies, the impact of exercise on different miRNAs has been explored. Melo and co-workers have discovered a key role of miR-214 in rats [5]. While this miRNA was down-regulated in the cohort undergoing exercise, the target gene SERCA2a increased. Similarly, Liu and co-workers demonstrated that miR-222 is of key importance for cardiac growth, which is induced by exercise. Furthermore miR-222 protects against pathological cardiac remodeling [6]. While these studies have been carried out in animals, case–control studies have been also conducted in human athletes. Wardle et al. have carried out a study on plasma of endurance and strength athletes [7]. They investigated three cohorts (strength athletes, power athletes and untrained individuals, n = 10) and found differential regulation of circulating miRNAs such as miR-222. A more elaborate overview describing the role of circulating microRNAs in response to exercise can be found in two recently published comprehensive reviews by Xu [8] and Altana [9].

Especially with increasing evidence of miRNA profiles for diagnostic or prognostic purposes in human pathologies putative confounding variables are also gaining increasing attention. Beyond straightforward confounders of miRNA profiles such as the age and gender [10] regular exercise on a long term as well as short term increase after fatigue (e.g. in competitions or training camps) may also impact the miRNA profile. Concordantly, Gomes and co-workers described the implications on clinical diagnostics of using microRNA-based biomarkers in exercise [11].

Despite multiple studies on circulating miRNAs related to fatigue status, it is hard to compare different training forms, athlete types and the variations in blood and plasma. In the available studies, different protocols for extracting the miRNAs, profiling them and evaluating them have been applied. This renders a meta-analytical analysis error prone. We thus set to implement a standardized repository of miRNAs related to physical exercise.

In our previous proof-of-concept study [4] we did not distinguished between strength and endurance athletes, but analyzed less trained individuals as controls. In addition, we analyzed whole blood only. In the present study, we selected a study set-up that allows for paired and unpaired comparison of the most important factors. In detail, we measured the full miRNomes (1) of strength athletes and endurance athletes (2) before and after a 6-days simulated training camp in (3) plasma and blood. With all combinations included we profiled a total of 8 different groups. Since the comparisons “prior and post training” and “plasma and blood” were done analyzing same individuals, it was possible to do a paired testing for the respective comparisons. An unpaired analysis was required only for the comparison between strength and endurance athletes. Altogether, we profiled 29 athletes corresponding to 29 biological replicates. For each athlete we measured the miRNomes of the following four profiles: “prior training plasma”, “post training plasma”, “prior training blood” and “post training blood”, totaling 116 miRNomes.

## Methods

### Design

Twenty nine well-trained male athletes [15 endurance athletes (cyclists) and 14 strength athletes] volunteered for this prospective, short-term training trial. Tests were conducted at baseline following a 2-day run in resting phase as well as after induction of fatigue by a discipline specific, strenuous training program. The 6-day training period consisted of two training sessions a day, with the exception of day 4 when no morning session was scheduled. Care was taken to implement a demanding, training design for either discipline resulting in high levels of physical strain and fatigue. To verify effective, reversible induction of fatigue established fatigue markers [12] were assessed.

The study was undertaken in accordance with the Declaration of Helsinki and approved by the local ethics committee (Ärztekammer des Saarlandes, Saarbrücken, Germany, ID 46/13). All athletes provided written informed consent prior to participation. All tests were conducted in a University department (Cyclists: Saarland University, Germany; Strength athletes: Ruhr Universität Bochum, Germany).

### Blood sampling and miRNA measurement

After reporting to the laboratory at a standardized time (between 8 and 10 a.m., intra-individually same hour for all tests) subjects rested in the supine position for 10 min prior to blood collection. A winged cannula was inserted into the antecubital vein during a short stasis (max. 30 s.). Serum and plasma aliquots were frozen at −80 °C within 60 min from blood collection and stored for later analysis. Whole-blood samples for the determination of miRNA expression were collected and stored in special tubes (PAXgene blood RNA tube, Becton–Dickinson, Germany). For miRNA measurement, blood and plasma samples have been used while further laboratory parameters (details below) were determined from serum samples. miRNA extraction of blood and plasma samples have been carried out as described previously and according to manufacturer’s instructions [4]. For the strength athletes, plasma has been diluted in a ratio of 1:2. The quality of the samples has been controlled by Bioanalyzer measurements and the RNA 6000 Nano kit. Microarray profiling has been carried out using Agilent SurePrint V16 Human miRNA Microarrays (miRBase v16) microarrays encompassing 1205 different miRNAs, as described previously [4] and following the manufacturer’s instructions. Each miRNA was measured for each patient by 40 on-chip technical replicates. The microarray data are available in Additional file 1: Table S1.

### Further laboratory parameters

As further blood parameters we included the hemoglobin concentration (Hb), Erythrocyte, Leucocyte and Thrombocyte count, creatine kinase (CK), urea, free-testosterone, c reactive protein (CRP), cortisol, glutamine (Gln) and glutamate (Glu) concentration, insulin like growth factor 1 (IGF-1), IGF-1 binding protein 3 (IGF-BP3), tumor necrosis factor (TNF), interleukin 6 (IL-6), and human growth hormone (HGH) at different time points.

### Statistical analysis

Following feature extraction using the Agilent’s image processing software with standard parameters, expression values were subjected to quantile normalization. All downstream statistical calculations have been carried out in R version 3.0.2. Principal Component Analysis has been done using the *prcomp* function, for ANOVA, the *aov* function was used. Hierarchical clustering was done using the Euclidian distance measure and complete linkage clustering by the *hclust* package. Visualization of heatmaps has been done by a modified version of the *heatmap.2* function.

Correlations of the identified miRNA to diseases were calculated using the downloaded version of the Human microRNA Disease Database (HMDD) (Version 2.0, data accessed on May, 28th, 2016) [13]. Before relating the miRNAs to the HMDD, mature forms were mapped to the precursors to ensure compatibility with the HMDD. We used only the “circulating” subset of the HMDD. Prediction of pathways has been carried out using the gene (miRNA) set enrichment tool miEAA [14] (http://www.ccb.uni-saarland.de/mieaa_tool/). KEGG pathway and gene ontology analysis has been performed using miRTargetLink [15] (http://www.ccb.uni-saarland.de/mirtargetlink) and the API to GeneTrail [16] and GeneTrail2 [17].

### Availability of data and supporting materials

All miRNA measurements with meta-data have been made freely available (Additional file 1: Table S1).

## Results

**Fig. 1:**
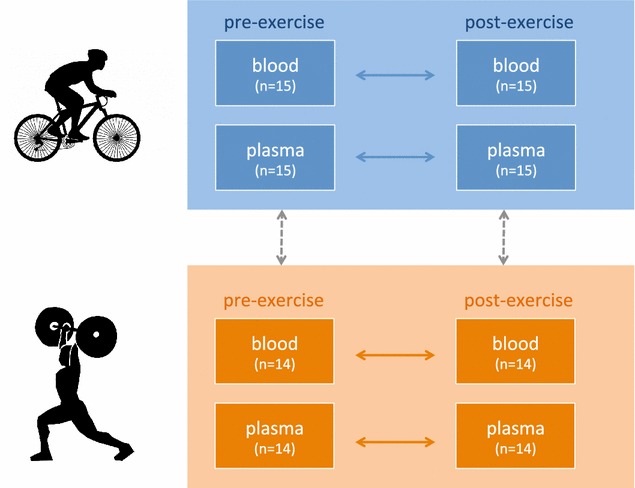
Study set-up. *Horizontal arrays* represent paired analyses, *vertical arrows* unpaired statistical calculations

To understand the influence of physical exercises on the miRNA abundance and to relate the affected miRNAs to known disease associated miRNAs we profiled 116 miRNOmes from 58 plasma and 58 paired blood samples of well-trained individuals (endurance and strength athletes) before and after a 6-day training period. The study-set up is sketched in Fig. 1. To limit the influence of markers close to the background and contributing more noise than signal to the analysis we removed low abundant miRNAs and performed the analysis with 388 miRNAs that were expressed in the majority of individuals. Effective induction of fatigue could be confirmed by changes in standard blood-born markers. Creatine kinase increased significantly in endurance and strength athletes [endurance: +56 ± 74 U/l (p = 0.006); strength +664 ± 817 U/l (p = 0.013)]. Urea increased significantly in endurance athletes (+11 ± 10 mg/dl p < 0.001). For strength athletes the difference in urea concentration failed to reach statistical significance [+4 ± 11 mg/dl (p = 0.184)].

### Difference in composition of miRNA profiles

**Fig. 2:**
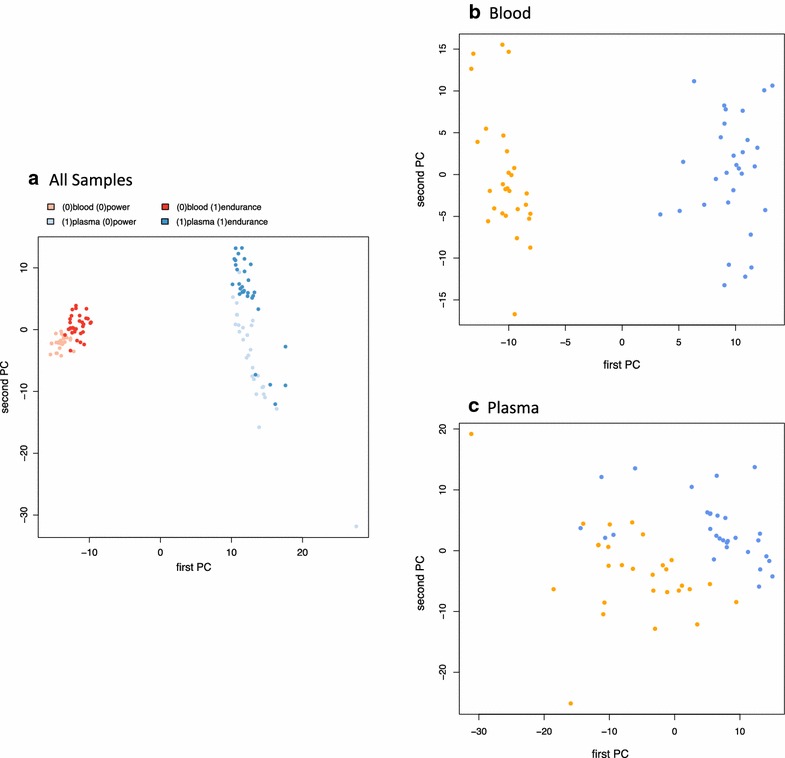
Principal component analysis. **a** presents PCA of all data points (*red* blood samples, *blue* plasma samples). **b** contains only blood and **c** only plasma samples (*orange* strength athletes, *blue* endurance athletes)

**Fig. 3:**
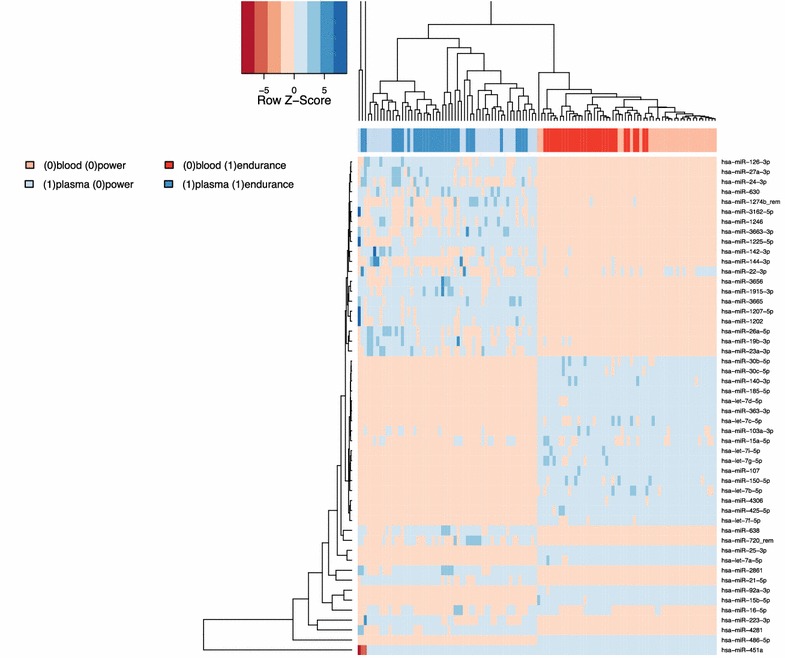
Hierarchical clustering of all samples. The *heatmap* indicates that substantial differences between blood and plasma are observed but also profiles between endurance and strength athletes generally cluster together

To understand how divergent miRNA profiles in blood and plasma of strength and endurance athletes are and how the current training and individual variations contribute to the profiles we quantified the effect of these four variables on the miRNA abundance. Principal variance component analysis highlighted that the specimen type (blood versus plasma) had the largest impact on the profiles followed by the athlete type (endurance versus strength athletes). Individual variations and the training effect contributed substantially less to the overall profiles. While the large deviation between blood and plasma miRNomes is known the differences between strength and endurance athletes was surprisingly high. For visualizing the effect of the two most important parameters we performed principal component analysis and colored the individual samples with respect to the specimens and the athlete type (see Fig. 2a). The first PC perfectly distinguishes between plasma and blood profiles while the second PC well separates endurance from strength athletes. The figure also indicates that the variability in plasma profiles exceeded the variability of blood profiles. Nevertheless, blood profiles differentiated more precisely between strength and endurance athletes. To demonstrate this we performed PCA separately for blood (Fig. 2b) and plasma (Fig. 2c), providing evidence that for the blood-borne miRNA profiles strength athletes can be perfectly separated from endurance athletes while in case of plasma profiles a limited overlap is observed. A similar conclusion can be drawn from an unsupervised hierarchical clustering, presented in Fig. 3. The heat map outlines a clear cut between blood and plasma profiles. For the blood profiles on the right hand side of this plot, almost all strength and endurance athletes cluster well together.

These results suggest that the type of training has a substantial impact on plasma and blood profiles of individuals. The miRNA profiles, especially from blood, can thus likely be used as molecular monitor for the strength and stamina of athletes.

### Specific miRNAs as indicators for different training modes

**Fig. 4:**
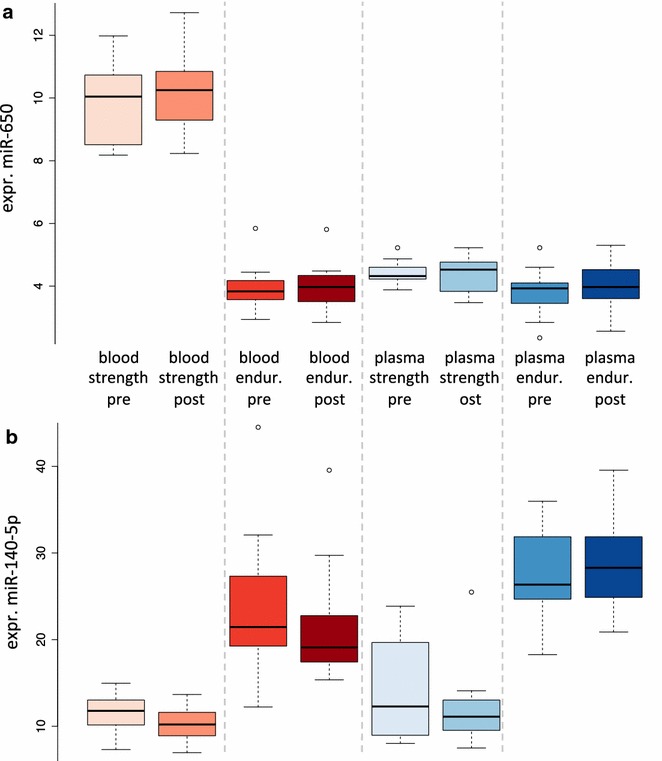
*Box* plots of two selected examples in the eight tested groups. **a** The first miR-650 is only expressed in blood of strength athletes. **b** The second miR-140-5p is up-regulated in blood and plasma of strength as well as endurance athletes

The described substantial variation between endurance and strength athletes in general let us ask on specific miRNAs that are differentially regulated in blood and plasma for the two athlete types. Following adjustment for multiple testing we observed in plasma samples 231 differentially regulated miRNAs. Of those, almost equal parts were higher (108 miRNAs)/lower (123) expressed in endurance athletes as compared to strength athletes. The most significantly down-regulated miRNA was hsa-miR-513b-5p, the most significantly up-regulated miRNAs was hsa-miR-140-5p, both with a p value of 3 × 10^−13^. In blood off the same athletes, 265 differentially regulated miRNAs following adjustment for multiple testing were detected. Of these 139 were higher expressed in endurance athletes while 126 were lower expressed. Most significantly down-regulated was hsa-miR-650 (p value of 4 × 10^−23^), most significantly up-regulated was hsa-miR-3620-3p (p value of 6 × 10^−19^). The expression of miR-650 is exemplarily presented in Fig. 4a. As detailed there, we calculated a slight correlation between the differential regulation of miRNAs between strength and endurance athletes in blood as well as plasma (Pearson Correlation of 0.16). Among the miRNAs that showed largest concordance was miR-140-5p, the most significantly up-regulated miRNA in plasma was likewise significantly up-regulated in blood of endurance athletes (Fig. 4b).

Taken together, our results suggest that miRNAs, preliminary measured from blood, are well suited to indicate the overall status of an athlete and whether the respective athlete is trained mostly towards endurance or strength.

### miRNA enrichment of markers depending on training mode

**Fig. 5:**
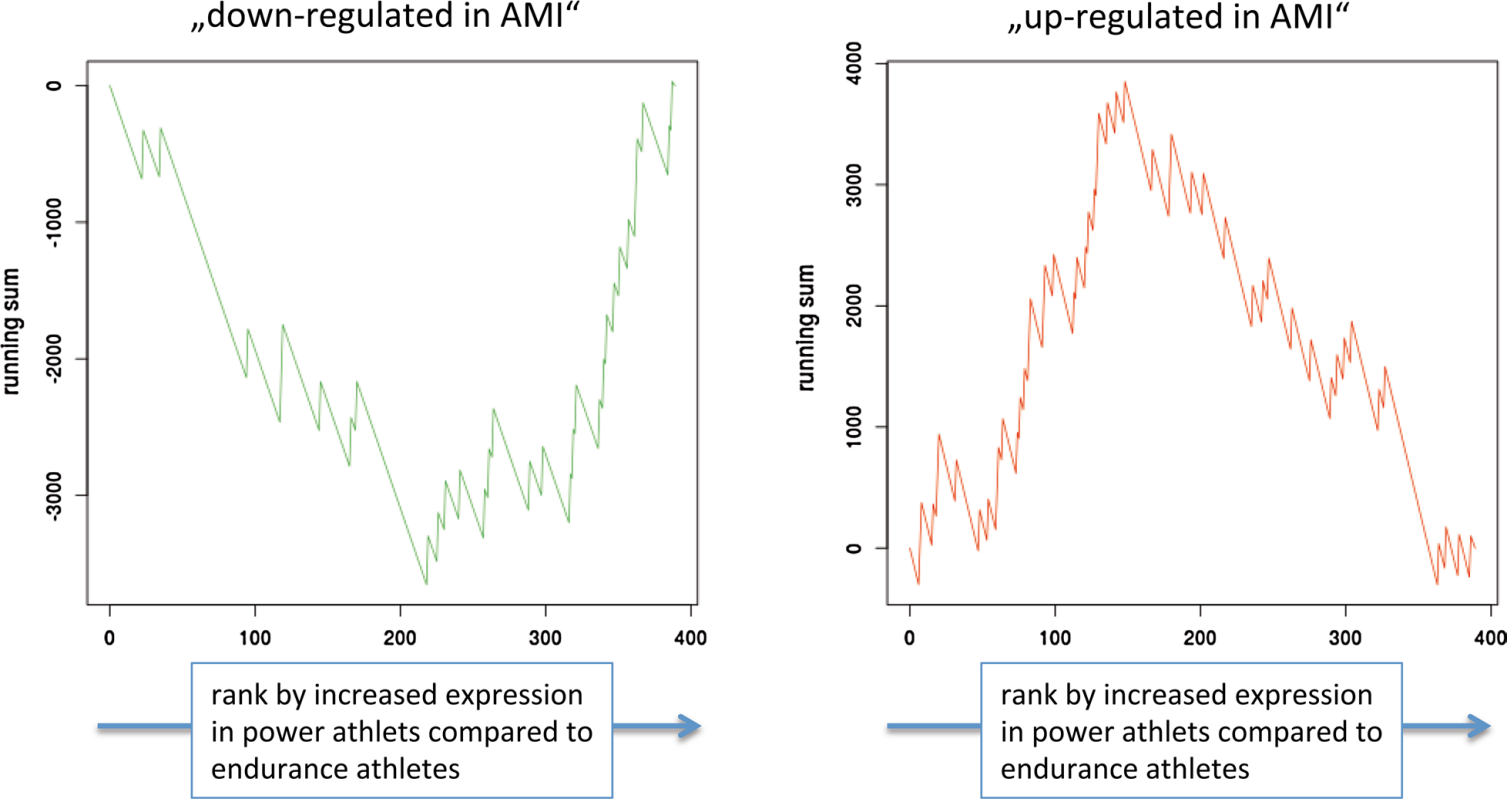
Running sum that highlights “acute myocardial infarction” miRNAs to be significantly changed in strength versus power athletes

In the light of the substantial changes in the miRNA repertoire of endurance and strength athletes we applied a statistical set enrichment analysis to understand on which pathways and biological categories the dys-regulated miRNAs participate using miEAA (http://www.ccb.uni-saarland.de/mieaa_tool/). In the miEAA data repository we also integrated data of more than 25 human pathologies to allows for detecting significant enrichment of disease markers in our miRNA sets. The calculations have been carried out for plasma and blood separately. With respect to disease associations, we calculated significant enrichment for miRNAs that are up- or down regulated in acute myocardial infarction (FDR adjusted significance value of 0.04 and 0.01, respectively), as the running sum statistics in Fig. 5 demonstrate. These plots show on the x-axis the rank of differential regulation (left miRNAs are up-regulated in strength athletes, right miRNAs in endurance athletes). The y-axis shows the running sum value, i.e. the higher or respectively lower the curve gets the more accumulation of miRNAs in the considered categories are observed. We also found that the miRNAs that are higher expressed in endurance athletes as compared to strength athletes are enriched in different immune cells (CD15: 2 × 10^−5^; CD56: 0.0001; CD3: 0.0002, CD14: 0.0003, CD19: 0.0008). For blood samples substantially less significant results were observed. Most notably, we calculated enrichment of miRNAs targeting ischemia genes in endurance athletes. This opens the question on the impact of miRNAs differentially regulated in endurance and strength athletes.

### Pathway analysis of miRNA targets

**Fig. 6:**
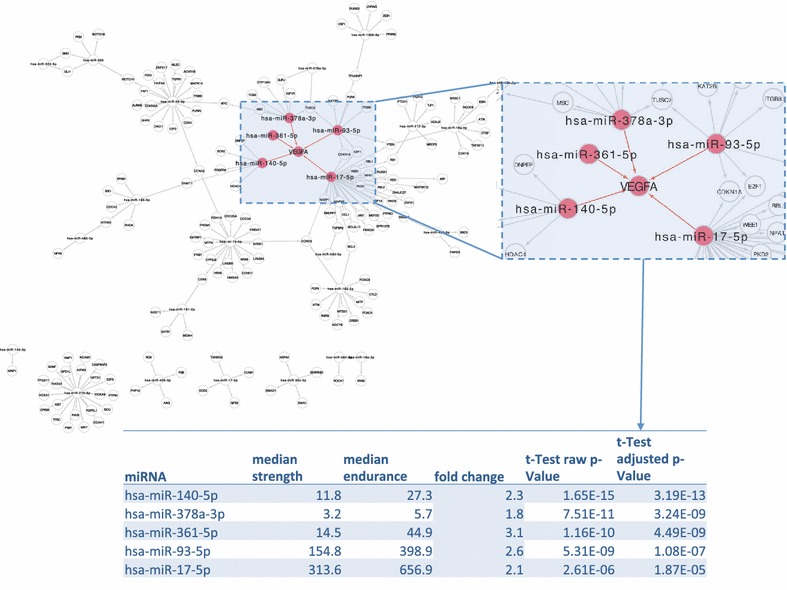
Network analysis of target genes. For miRNAs dys-regulated between strength and endurance athletes we mapped target genes by functional experimental analysis. The most central gene was VEGFA, which is regulated by five miRNAs. As the *table below* the figure highlights, all miRNAs targeting VEGFA are up-regulated in endurance athletes

Dys-regulation of miRNAs should lead to a dys-regulation of target genes that in turn might have an influence on the phenotype. Following this hypothesis we extracted target genes of miRNAs that are differentially regulated in plasma of strength and endurance athletes. To select the most relevant regulators we selected miRNAs with adjusted significance value below 10^−4^ and included only experimentally validated targets. For the 63 dys-regulated miRNAs we extracted 189 targets. Some of these have been targeted by different miRNAs, leaving 162 unique genes. The respective miRNAs and target genes are sketched as interaction network in Fig. 6. A network analysis aiming to discover the most central gene (i.e. a gene which is targeted by many miRNAs) highlighted VEGFA. The respective part of the network is also presented in Fig. 6. VEGFA is targeted by five miRNAs, the previously mentioned miR-140-5p and also miR-378a-3p, miR-361-5p, miR-93-5p and miR-17-5p. All these miRNAs are significantly up-regulated in endurance athletes compared to strength athletes (1.8 fold to 3.1 fold).

To further annotate the genes in the network we carried out functional gene enrichment analysis. We compared the accumulation of the 162 unique miRNA targets on KEGG pathways and Gene Ontology categories to the background distribution of all genes that are targeted by at least one miRNA with strong evidence (2098 genes). Following adjustment for multiple testing according to Benjamini-Hochberg and excluding categories with a less than three-fold enrichment of genes, we found 8 significant categories from the gene ontology (biological process) and ten KEGG pathways. Remarkably, the first two categories were correlated to diseases, including the KEGG pathways “Melanoma” (4.6 times more genes than expected, p = 3.1 × 10^−6^) and “Bladder cancer” (5.4 times more genes than expected, p = 1.4 × 10^−5^). The third most significant category was the KEGG pathway “Cell Cycle” (3.6 times more genes than expected, p = 1.8 × 10^−5^). The previously described gene VEGFA was not only contained in disease pathways but also in the Gene Ontology category “cell maturation” and the KEGG pathway “PI3 K-Akt signaling pathway”.

### Specific correlation of miRNAs to diseases

To find correlations of miRNAs to diseases we queried the Human miRNA and disease database (HMDD) as described in the “Methods” section. Importantly, we only used unique combinations of miRNAs to diseases in the “circulation” data set from the HMDD, that includes 512 associations for 240 miRNAs. Altogether, we found 74 matches, i.e. 74 of the 512 entries in the HMDD contained one of the 63 dys-regulated miRNAs. Almost 50 % of the 63 miRNAs in turn were associated with at least one disease. The largest number of associations was found for hsa-mir-17, being correlated with 18 cancerous disorders. The associations between miRNAs and diseases are summarized in Additional file 2: Table S2. The results indicate that the training mode potentially influence disease diagnosis by circulating miRNAs.

### Specific miRNAs as indicators for fatigue

**Fig. 7:**
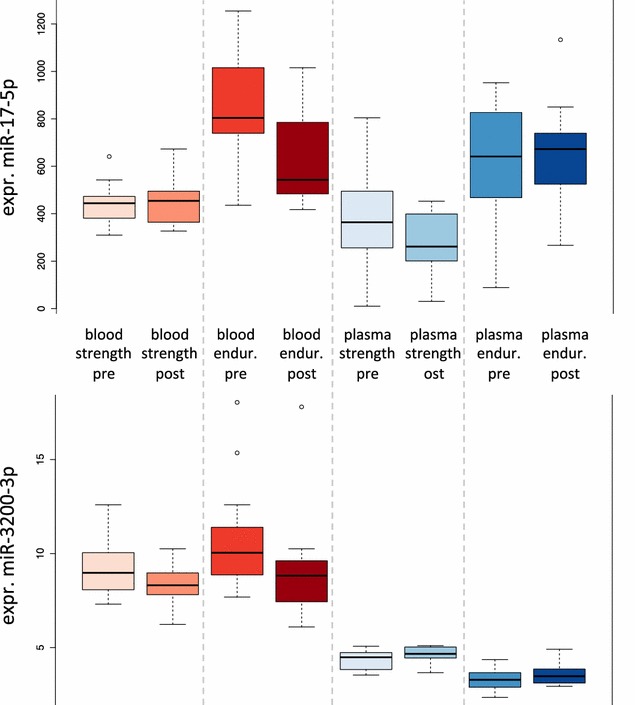
Effect of training. The provided examples indicate that also short-time influences of miRNAs to training are observed

Although the 6-day training period itself seems to have only a limited influence on the miRNA profiles in total, specific miRNAs can potentially be altered in their abundance by the fatigue. To quantify the effect of training we separately compared blood and plasma samples for endurance and strength athletes such that a total of four paired comparisons has been carried out: (1) plasma of strength athletes pre/post training (2) blood of strength athletes pre/post training (3) plasma of endurance athletes pre/post training and (4) blood of endurance athletes pre/post training. To compare the results better to each other, raw p values have been taken into consideration. Of 388 miRNAs, 24 were significant in comparison 1 (6.2 %), 56 in comparison 2 (14.4 %), 21 in comparison 3 (5.4 %) and 57 in comparison 4 (14.7). While the results for plasma miRNAs are close to the expected value of 5 % for the alpha level of 0.05, we observed an increased fraction of significant miRNAs for the comparison in blood of endurance as well as strength athletes. The effects in blood were such substantial that despite the small cohort and the large number of features miR-17-5p remained significant even after correction for multiple testing in endurance athletes. Remarkably, this miRNA was also among the most dys-regulated between strength and endurance athletes and its precursor was associated to 18 diseases in the HMDD. The expression of miR-17-5p is presented as box whisker plot in Fig. 7. Likewise, this figure presents the expression of miR-3200-3p, which is most significantly down-regulated following the training period in strength and endurance athletes. The ten most significant miRNAs (minimum p value in any of the four comparisons) are summarized in Table 2. As presented in Table 1, all significantly changed miRNAs in blood following training are lower abundant as compared to the baseline value.

**Table 1:** KEGG and GO enrichment analysis

Category	Database	Target genes on category	Expectation value	Enrichment	p value
Melanoma	KEGG	17	3.7	4.6	3.10E−06
Bladder cancer	KEGG	13	2.4	5.4	1.36E−05
Cell cycle	KEGG	19	5.3	3.6	1.83E−05
Glioma	KEGG	15	3.4	4.4	1.83E−05
G1_phase(4) & mitotic_G1_phase(5)	GO	11	1.3	8.3	2.34E−05
Viral carcinogenesis	KEGG	20	6.0	3.3	2.59E−05
Pancreatic cancer	KEGG	16	4.1	4.0	2.72E−05
Chronic myeloid leukemia	KEGG	17	4.6	3.7	3.04E−05
Non-small cell lung cancer	KEGG	12	3.1	3.9	5.86E−04
p53 signaling pathway	KEGG	11	3.2	3.4	3.44E−03
Thyroid cancer	KEGG	6	1.4	4.3	2.67E−02
Biological_phase(2)	GO	15	4.9	3.1	4.68E−02
Cell_cycle_phase(3)&mitotic_cell_cycle_phase(4)	GO	15	4.6	3.3	4.68E−02
Cell_maturation(5)	GO	14	4.2	3.3	4.68E−02
Regulation_of_fibroblast_proliferation(5)	GO	12	3.3	3.6	4.68E−02
Positive_regulation_of_fibroblast_proliferation(5)	GO	10	2.3	4.3	4.68E−02
Interphase(4)	GO	9	1.9	4.8	4.68E−02
Hair_follicle_morphogenesis(4)	GO	7	1.2	6.0	4.68E−02

Significant categories containing at least three times more genes than expected by chance. Provided are the category name, the database where data have been extracted, the number of targets genes of the 63 miRNAs of this category, the expected number of genes given the 2098 background genes, the enrichment factor, and the p value following adjustment for multiple testing

**Table 2:** Pair-wise comparisons of prior- and post training for endurance and strength athletes in blood and plasma

miRNA	Plasma strength p	Plasma strength AUC	Blood strength p	Blood strength AUC	Plasma endurance p	Plasma endurance AUC	Blood endurance p	Blood endurance AUC
hsa-miR-17-5p	0.1017	0.39	0.7784	0.53	0.8919	0.47	*0.0001*	0.26
hsa-miR-454-3p	0.4262	0.52	0.5771	0.55	0.9280	0.48	*0.0003*	0.33
hsa-miR-20a-5p	0.3969	0.43	0.7346	0.44	0.8167	0.56	*0.0005*	0.29
hsa-miR-590-5p	0.1679	0.39	*0.0007*	0.27	0.4055	0.58	0.2603	0.36
hsa-miR-3200-3p	0.1099	0.67	*0.0009*	0.35	0.3392	0.59	*0.0064*	0.26
hsa-miR-29c-3p	*0.0363*	0.67	*0.0010*	0.34	0.6568	0.55	0.7085	0.50
hsa-miR-27b-3p	0.8400	0.52	0.3308	0.54	0.2059	0.57	*0.0013*	0.27
hsa-miR-513a-5p	0.7051	0.54	0.3726	0.42	*0.0014*	0.25	0.1464	0.60
hsa-miR-26b-5p	0.3362	0.41	0.1040	0.65	0.4129	0.58	*0.0014*	0.31
hsa-miR-1246	*0.0019*	0.86	0.9294	0.60	0.2875	0.55	0.6481	0.59

The significance values are shown along with the AUC values. Significant findings are highlighted in italics

### Correlation of miRNAs to other laboratory parameters

The athletes participating in this study were characterized for other common blood parameters as detailed in the “Methods” section. We correlated all miRNAs to all clinical parameters in a pair wise manner using Spearman correlation. To correct for multiple testing we applied the Bonferroni correction to minimize the number of false positive hits. Following this adjustment, 14 correlations of miRNAs with blood parameters remained significant. We found three correlations of the creatine kinase (CK) at day 5 with miR-18-3p, miR-33b-3p or miR-650. Both miR-1305 and miR-3198 correlated with IGF-BP3 after training and miR-128-3p correlated with the free testosterone. MiR-140-5p in plasma was negative correlated to hemoglobin while plasma miR-188-5p was positive correlated to hemoglobin.

## Discussion

Levels of circulating miRNAs are not only altered in pathological processes but are also affected by training condition. In this study we examined the distribution of miRNAs in blood and plasma of strength and endurance athletes before and after training. The influence of physiological processes, such as physical exercises, on the profiles of circulating miRNA may confound disease diagnosis by miRNAs potentially limiting the translation of miRNA profiles to clinics [18].

First, we provide evidence that miRNAs in blood and plasma are different between strength and endurance athletes and are also indicative for the overall fatigue status of competitive athletes. The effect sizes of differences in blood outreach the differences in plasma clearly. Fatigue induced changes were only detectable when using a repeated measures approach, pointing to the need for an individualized interpretation of measured values. Moreover, we provide evidence that longitudinal measurement of blood miRNAs may add to individualized and personalized training.

Different miRNAs have been described as markers for fatigue status or altered between strength and power athletes (see “Background” section and [3, 9]). The respective miRNAs were also observed in our study. However usually other markers exceeded the effect sizes of the known markers substantially. This may be due to different factors. First, our study profiles over 12,000 miRNAs that are annotated in the version 16 of the miRBase (http://www.mirbase.org). Second, we carried out a paired study set-up and no unpaired case–control study. Third we measured more individuals as most other studies. Altogether, for 29 athletes four different full miRNomes were profiled, resulting in 116 profiles.

By investigating signaling cascades we discovered a high regulatory influence of miRNAs that are dys-regulated in strength and endurance athletes on several genes. Most significant was the regulatory influence of miRNAs on a vascular endothelia growth factor (VEGF), namely VEGFA. Increased capillary density in skeletal muscle is a well-known effect of exercise training which promotes oxygen supply during exercise by increasing diffusion area and reducing diffusion distance. Angiogenesis has been observed with various exercise modes including endurance [19] and strength training [20]. However an increase in capillary density is generally observed with endurance training only, reflecting the requirements of aerobic energy production [21]. VEGF is a key pro-angiogenic regulator of capillary growth in response to physical exercise [22]. Therefore, at first sight, the higher expression of miRNAs which (down-) regulate VEGF seems surprising. However, capillary growth in response to exercise training is a highly regulated process involving a wealth of pro- as well as anti-angiogenic factors [21]. Two of the most important regulators promoting VEGF expression during exercise are AMP-activated protein kinase (AMPK) [23] and the transcriptional co-activator PGC-1α [24], which are also fundamental for the adaptation to endurance training [25]. Therefore, avoiding excessive angiogenesis caused by the targeted activation of these pathways with competitive endurance training seems to be a plausible explanation for this finding.

Besides, it is important to understand the variability of miRNAs dependent on the training (including long term effects but also short term effects) since miRNAs are important disease markers. This in turn means that intensive, long-term training or even one single exhaustive event may confound disease diagnosis substantially. Similar effects are e.g. known for cardiac markers such as cardiac troponin and BNP [26, 27]. As shown in the “Results” section, many miRNAs are associated with different diseases. Building on these results we investigated miRNA changes as result of the training condition. We carried out a comprehensive literature research for the most significantly changed markers in blood, serum and plasma, including 46 markers that had adjusted p-values of below 10^−10^ in any of the two comparisons. The most significantly altered miR-650 was correlated to a range of different pathologies, including heart failure [28], congenital heart disease [29], diabetic ischemic heart failure [30] and different cancer types (gastric cancer [31], melanoma [32], leukemia [33], hepatocellular carcinoma [34], glioma [35], colorectal cancer [36] and lung cancer [37]). Other key miRNAs included miR-221-3p as marker for Non-ST-Segment and ST-Segment-Elevation Myocardial Infarction [38] and stroke [39]. In the latter publication another miRNA significantly affected in our study was described: miR-140-5p. The same holds for miR-221-3p and miR-98-5p in preeclamptic patients [40]. Another study highlighted two miRNAs (again miR-140-5p as well as miR-532-5p) linked to type 2 diabetes that change with insulin sensitization [41]. The same two miRNAs have been found as markers for obesity [42]. Beyond these examples we extracted 231 hits for the 47 miRNAs (for 31 miRNAs at least a single hit was found) from PubMed (http://www.ncbi.nlm.nih.gov/pubmed) in 211 different publications.

The overall high correlation of miRNAs affected by training to diseases provide evidence that the training condition is an important confounding variable for miRNA biomarker studies that has to be taken into account in developing diagnostic and prognostic signatures.

## Conclusion

This study represents the most comprehensive miRNA atlas of athletes to date. Our study set-up that includes four measurements per individual athlete allowed for quantifying differences in strength and endurance athletes, in serum and plasma and before and after a 6 days training camp.

Overall, we report a limited immediate influence of the 6 days training on the miRNA profiles overall but describe substantial differences between profiles of strength and endurance athletes. miRNAs especially expressed in endurance athletes but less expressed in strength athletes significantly targeted VEGFA. These differences in blood by far exceeded the differences in plasma, which have already been described. Additionally, we provide evidence that miRNA profiles prior to exercise correlate very well with measurements of creatine kinase carried out following 5 days of physical training.

Finally, we describe that the miRNAs that are affected by exercising according to our results are important disease markers. For a diagnosis or prognosis of human pathologies the amount and kind of physical training conducted prior to blood sampling is in consequence a very important confounding variable.

## Additional files

10.1186/s12967-016-0974-x

10.1186/s12967-016-0974-x

## Abbreviations

PCA: principal component analysis
miRNA: microRNA
VEGFA: vascular endothelial growth factor A
cTnT: cardiac troponin T
BNP: B-type natriuretic peptide
CRP: c reactive protein
Gln: glutamine
Glu: glutamate
IGF-1: insulin like growth factor 1
IGF-BP3: IGF-1 binding protein 3
TNF: tumor necrosis factor
IL-6: interleukinn 6
HGH: human growth hormone
CK: creatine kinase
