# Erratum to: miRNAs and sports: tracking training status and potentially confounding diagnoses

**DOI:** 10.1186/s12967-016-1054-y

**Published:** 2016-10-13

**Authors:** Anne Hecksteden, Petra Leidinger, Christina Backes, Stefanie Rheinheimer, Mark Pfeiffer, Alexander Ferrauti, Michael Kellmann, Farbod Sedaghat-Hamedani, Benjamin Meder, Eckart Meese, Tim Meyer, Andreas Keller

**Affiliations:** 1 Institute of Sports and Preventive Medicine, Saarland University, Saarbrücken, Germany; 2 Department of Human Genetics, Saarland University, Saarbrücken, Germany; 3 Chair for Clinical Bioinformatics, Medical Department, Saarland University, Building E2.1, 66125 Saarbrücken, Germany; 4 Department of Theory and Practice of Sports, Johannes Gutenberg University of Mainz, Mainz, Germany; 5 Faculty of Sport Science, Ruhr-University Bochum, Bochum, Germany; 6 School of Human Movement Studies, The University of Queensland, St Lucia, Australia; 7 Internal Medicine, Heidelberg University, Heidelberg, Germany

## Erratum to: J Transl Med (2016) 14:219 DOI 10.1186/s12967-016-0974-x

Unfortunately, the original version of this article [1] contained an error. The name of the author [Farbod Sedaghat] was incorrect, the author’s surname should be Sedaghat-Hamedani. The author’s name is Farbod Sedaghat-Hamedani.
